# Effect of landscape irrigation regulations on runoff volume from low-density residential catchments during two California droughts

**DOI:** 10.1016/j.heliyon.2023.e23602

**Published:** 2023-12-15

**Authors:** Zhou Yang, Lorence R. Oki, Jared A. Sisneroz, Darren L. Haver, Bruno J.L. Pitton

**Affiliations:** a Department of Plant Sciences, University of California, Davis, CA, 95616, USA; b University of California South Coast Research and Extension Center, Irvine, CA, 92618, USA

**Keywords:** Water conservation, Irrigation regulation, Dry season runoff, Urban runoff

## Abstract

The severe drought in California (2012–2016) generated significant public and government concern. State and local watering regulations were enacted to reduce residential and commercial water-use during the droughts. This study presents a comparison of residential runoff volumes before and after local landscape irrigation regulations were enacted during the droughts of 2008 and 2012–2016. Each sampling site (Folsom 1 and Folsom 2) was a storm drain outfall that drained a low-density residential catchment in the City of Folsom. Dry season runoff measured at the sampling sites represents neighborhood outdoor water waste, mainly from landscape irrigation. During the drought of 2012–2016, median runoff flows were significantly reduced after local landscape irrigation regulations were enacted. The daily runoff pattern was also highly influenced by regulation, with reductions of daily peak runoff flows on 4–5 days in a week after watering regulations were enacted. The number of peak flow events in the daily runoff pattern were reduced during this period. In addition, a significant reduction in mean runoff volume occurred. Based on these results, the watering regulations enacted by the City of Folsom had a positive effect on reducing urban runoff from residential neighborhoods during the dry season. As the results are from monitoring sites in a relatively small geographical area, further work should evaluate reductions in irrigation runoff from other California locations to determine if this is a localized phenomenon.

## Highlights

• During short-term drought, irrigation regulations resulted in reductions in runoff.
• Dry season runoff declined during long-term drought without irrigation regulations.
• Irrigation regulations produced more reduction in runoff during long-term drought.
• Runoff magnitude and frequency decreased after irrigation regulations were enacted.

## 1 Introduction

Following the 1997–1998 El Niño event, nearly the entire United States underwent a drought that lasted until the fall of 2002. This so-called “drought of the century” returned in the winter of 2004 and persisted in the US Southwest, with California experiencing extreme drought during that period [1,2]. Due to its Mediterranean summer-dry climate, California relies heavily on winter storms to generate snowpack in the Sierra Nevada mountain range. The resulting snowmelt supplies surface water to streams and lakes during the late spring and summer [3]. Precipitation in the Sacramento and San Joaquin river basins was below normal for 10 of the 14 years from 2001 to 2015 [4]. During this same period, water demand grew, straining limited resources and generating considerable interest in water conservation [5,6].

To reduce the risk of water shortages, California municipalities responded to drought by implementing management measures [7] including restricting nonessential water use, increasing water supply through ground water pumping, and encouraging water conservation and recycling. Measures enacted by the City of Folsom, CA, USA, the location of this study area, focused on reducing landscape irrigation by supporting efficient irrigation practices to reduce water waste. In 2015, California Governor Brown also ordered the State Water Resources Control Board to reduce potable urban water use by 25 % from 2013 water use [8].

Landscape water runoff during dry periods can result from poor irrigation practices, including over application and poor irrigation system distribution uniformity. Since in many parts of California, little to no rainfall occurs from June to September, urban runoff in the summer can be assumed to result solely from anthropogenic activities such as landscape irrigation and car washing. Urban runoff is identified as a leading source of water quality issues [9,10]; it also presents a problem in water conservation. The adverse impact of urban runoff to water quality has been well studied, as nutrients, pesticides, and other constituents of concern are carried from urban landscapes to natural areas, polluting water bodies downstream [11,12].

This situation is exacerbated in drought years as there is little precipitation to dilute pollutants in the runoff that contaminate surface and ground waters. However, studies of urban runoff during droughts have focused on the concentrations of pollutants in the runoff [10,11,13]. To the authors’ knowledge, no research exists related to the volume of urban runoff during droughts and the effect of water-use regulations that are imposed in response to drought. Determining if landscape water-use regulations are effective at reducing landscape water use could be valuable to policy-makers and inform regulations regarding water conservation during drought.

This paper describes urban runoff patterns during the dry season from two monitored sites in Northern California before and after local landscape irrigation regulations were implemented during two drought periods. We will address the following questions.

1. Do local mandatory landscape irrigation regulations reduce urban water runoff volumes during the dry season?
2. Does imposition of mandatory landscape irrigation regulations change daily urban runoff flow patterns during the dry season?
3. Was a long-term (Dec 2013–May 2016) landscape irrigation regulation more effective in reducing urban runoff during the dry season compared with a short-term regulation only during 2008, a single drought year?

To address these questions, we investigated urban runoff data collected during the dry season from two monitored sites in the City of Folsom, CA during two drought periods (2008 and 2012 to 2015). The results were compared and assessed using statistical analysis and by mapping residential area runoff flows.

## 2 Materials and methods

### 2.1 Samples collection

Flow from storm drains was monitored from January 2008 to December 2015 within two low-density residential areas of single-family homes in the City of Folsom, Sacramento County, California, USA; these sites were labeled Folsom 1 and Folsom 2. The neighborhoods were selected based on the following criteria: they consist only of single-family residences that are of similar development age, lot size, and assessed value. The monitoring sites were selected based on sample collection viability. The surface runoff from each of the residential catchments drained to separate single storm drain outfalls where flows were measured and samples were collected. Details of the site selection process [14] and pollutant load estimates for Folsom 2 were described previously [10,15].

Study periods for this investigation were selected based on criteria of the year(s) of drought and the implementation of different watering regulations listed in the Folsom Water Conservation Ordinance No. 1118 [16]. The drought of 2008 was considered over on the day before the first moderate rainfall (>0.26 cm h^−1^) of the wet season [17]. In California, most rainfall occurred from November to February with only minimal amounts of rainfall occurring from May to October.

The Folsom Water Conservation Ordinance No. 1118 listed five water conservation stages to respond to different drought situations: Stage one (Basic Stage) included prohibitions on runoff from landscapes and requiring landscape irrigation from 10 a.m. to 10 p.m. This stage was in effect at all times of the year during the study periods unless a more restrictive conservation stage took effect. Stage two (Water Alert) restricted landscape irrigation to a maximum of three days per week based on an odd/even house number schedule. Stage three (Water Warning) limited landscape irrigation to a maximum of two days per week and also based on and odd/even house number schedule. Stage 1, 2, and 3 water conservation measures had been implemented during the droughts of 2008 and 2012–2015. In 2008, water conservation Stage 2 started on August 28th and ended in year 2010. During the drought of 2012–2015, Stage 3 took effect on Dec 13, 2013 and ended on May 13, 2016.

From the above criteria, two drought periods were selected for study. The first occurred in 2008 and consisted of two phases: 1) before water conservation regulation was enacted: June 1st to August 27th and 2) during regulation: August 28th to October 28th. The second drought occurred from 2012 to 2016 and included three phases: 1) before regulation: June 1, 2012 to September 30, 2013, 2) during regulation: June 1, 2014 to September 30, 2015, and 3) after regulation was downgraded to Stage 1: June 1 to September 30, 2016. Funding for data collection at Folsom 1 ended in 2015, hence only Folsom 1 data collected during the 2008 drought was used in this study.

At each site, storm drain outfalls consisted of 122 cm (48 inch) concrete pipes with sensors installed to record water pH, electrical conductivity, temperature, depth and velocity [15] and connected to Hach Sigma 950 Flow Meter dataloggers (Hach Corporation, Loveland, CO). Readings were collected every minute, and the mean of the readings were recorded every 2 min in 2008 and every 15 min in 2012–2016. Based on the water depth and velocity and the pipe geometry, long-term relationships between flow and water depth at each site were described by Refs. [14,18]:

Folsom 1: Q = 0.2234 × D^2^ - 1.3945 × D + 1.6989

Folsom 2: Q = 0.0351 × D^2.348^ (D < 5.2)

Q = 0.215 × D2 - 1.031 × D + 1.225 (D ≥ 5.2)

Where Q = Flow (L s^−1^).

D = Water Depth (cm).

### 2.2 Data cleaning

Data cleaning included discovery of errors occurring in equipment operation and recording. Simple outliers resulting from an error in measuring water depth were defined as water levels recorded with values lower than 0 cm or greater than 122 cm (outfall pipe diameter).

Continuous outliers that occurred longer than 30 min and less than 2 days were removed. Individual outliers that were not continuously recorded for more than 30 min were replaced with the estimated water depth. Replacement of an individual outlier was estimated by:

O = mode (O_-1_ - q_0.5,_ O_-1_ + q_0.5_)

Where O is individual outlier, O_-1_ is the sample before O, q_0.5_ is 50th percentiles of the water depth residuals.

Outliers continuously recorded for more than two days were visually reviewed. Long-term sample deviation with consistent variance was considered associated with machine operation error and corrected by recalibration of the base water depth. These outliers were corrected by subtracting the offset, where the offset is the difference between the baseline of the long-term outlier and the baseline of the flow water depth. Both baselines were visually determined in line graphs. Other continuous outliers with inconsistent variance were removed.

In order to apply consistent statistical methods to samples from both study periods, a continuous 30-min water-depth record was created by averaging 2-min or 15-min water depths within every 30-min period. The water flow and depth relationship curve was applied to the continuous 30-min water depth record to generate flow data for both sites.

### 2.3 Statistical analysis

Both statistical analyses and graphical displays were performed using R 3.4.3. Kolmogorov Smirnov (KS) tests evaluated whether the flow samples were drawn from the same distribution. An uneven *t*-test was used to determine whether the flow means before and during watering regulations were the same for each sample site. ANOVA and Tukey Honest Significant Difference tests were used for the 2012–2016 drought data to determine whether the significant difference of flows before and after regulations was caused by drought or regulation. Flow values for 2008 were log transformed prior to conducting the *t*-test, ANOVA test, and HSD test to meet the assumption of normality and flow values of 2012–2016 were Box-Cox transformed. A significance level of 0.05 was used in all statistical tests. The average runoff flow for each 4-h time interval during each day of the week were represented in heatmaps to display changes in urban runoff patterns in the dry season due to water use regulation.

## 3 Results

### 3.1 Mean runoff flows

Flow data was analyzed according to the status of regulation. The mean flow before regulation was significantly larger (p < 0.001) than the mean flow during regulation at Folsom 1 (Table 1). The median and maximum flow before regulation was also greater than the flow after regulation was increased to stage 2. However, statistical tests were not conducted to determine if these differences in median and maximum flow were significant.

**Table 1:** Runoff flows from Folsom 1 in 2008 before and during implementation of landscape irrigation regulations.

Period	Regulation status	Mean (L s^−1^)	Median (L s^−1^)	Max (L s^−1^)	p-value^a^
**Jun. 1 – Aug. 27, 2008**	Before	3.81	3.27	259.69	–
**Aug. 28 – Oct. 28, 2008**	During	2.73	1.84	248.83	***

a. p-value represents *t*-test results of the difference in mean flows between the current period and the previous period after log transformation. (***) indicates significance at p < 0.001.

The mean flow during the drought 2008 at Folsom 2 was similar before and during regulation (Table 2). The *t*-test result suggests that the mean of the transformed flows before regulation was significantly smaller than the mean during regulation (p < 0.001). However, the median flow during regulation was smaller than the median flow before regulation. The maximum flow during regulation was larger than before regulation was enacted.

**Table 2:** Runoff flows from Folsom 2 in 2008 before and during implementation of landscape irrigation regulations.

Period	Regulation status	Mean (L s^−1^)	Median (L s^−1^)	Max (L s^−1^)	p-value^a^
**Jun. 1 – Aug. 27, 2008**	Before	0.894	0.577	67.7	–
**Aug. 28. – Oct. 28, 2008**	During	0.909	0.086	320.8	***

a. p-value represents *t*-test results of difference of mean flows between the current period and the previous period after log transformation. (***) indicates significance at p < 0.001.

During drought years 2012–2016, mean urban runoff dry season flows at Folsom 2 were significantly different before and during regulation (p < 0.001), as well as during and after regulations (p < 0.001) (Table 3). The mean flow during regulation was lower than both the mean flow before and after the regulation was lifted. The greatest runoff flow during the 2012–2016 drought was before regulation was enacted (2012–2013) and the maximum flow declined in response to regulation similarly as the mean flow. However, the continued decline of the median flow over the three time periods (Table 3) indicates the distribution of flows changed over time.

**Table 3:** Runoff flows from Folsom 2 in 2012–2016 before, during, and after irrigation regulations.

Period	Regulation status	Mean (L s^−1^)	Median (L s^−1^)	Max (L s^−1^)	p-value^a^
**2012–2013**	Before	2.144	0.604	71.005	
**2014–2015**	During	0.302	0.016	46.792	***
**2016**	After	1.404	0.003	54.530	***

a. p-values represent Tukey HSD test results of differences between current and previous period mean flows after Box-Cox transformation with λ = 0.101. (***) indicates significance at p < 0.001.

The significant differences in mean flow between each period indicate that low-density residential urban runoff flows during the dry season were affected during the period when regulation was enacted. In order to further analyze whether watering regulation effect on runoff, flows during the drought were analyzed annually.

**Fig. 1:**
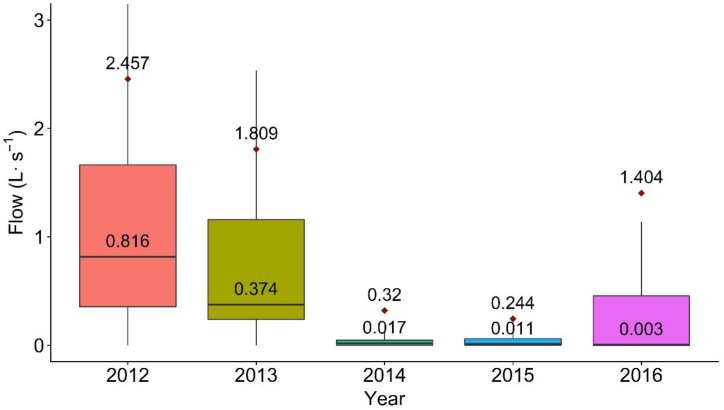
Box plots of annual runoff for each year from 2012 to 2016 at Folsom 2. Whisker lengths bounded by 1.5*IQR (interquartile range) and diamond symbol represents the mean value. Upper and lower value on each boxplot is mean and median, respectively.

The mean flow for each year was significantly different from the previous year (p < 0.001 for all years) (Fig. 1 and Table 4). These analyses show that runoff was reduced during drought, with or without watering regulation, and the year-to-year runoff reductions were consistent during drought from 2012 to 2015. The greatest mean reduction of runoff occurred between year 2013 and 2014. However, the smallest mean runoff reduction occurred between 2014 and 2015, the second year of local regulation. Runoff flows increased after the drought and the termination of irrigation regulation.

**Table 4:** Annual runoff flows from Folsom 2 before, during, and after implementation of landscape irrigation regulations.

Year	Regulation status	Mean (L s^−1^)	Median (L s^−1^)	Max (L s^−1^)	Difference (L s^−1^)^a^	p-value^b^
**2012**	Before	2.457	0.816	54.578	–	–
**2013**	Before	1.809	0.374	71.005	0.648	***
**2014**	During	0.320	0.017	46.792	1.489	***
**2015**	During	0.281	0.011	40.864	0.039	***
**2016**	After	1.404	0.003	54.530	−1.123	***

a. Difference = change in the mean flow for the year compared to the previous year.

b. p-values represent the Tukey HSD test results of differences between current and previous period mean flows after Box-Cox transformation with λ = 0.101. (***) indicates significance at p < 0.001.

### 3.2 Pattern of flow distribution

**Fig. 2:**
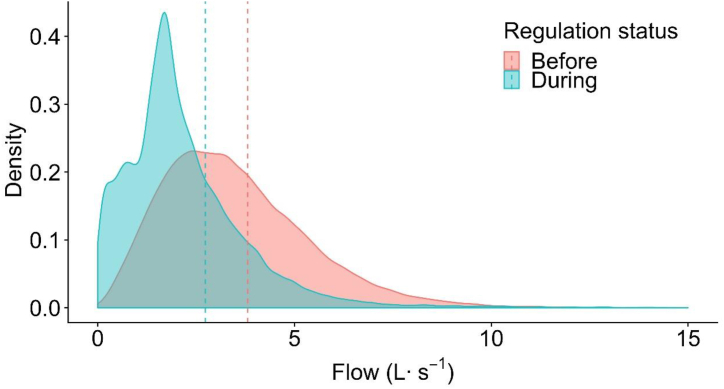
Density curve showing the relative frequency of runoff flow for 2008 at Folsom 1 before (June 1 – Aug. 27) and during (Aug. 28 – Oct. 28) landscape irrigation regulations. Vertical dashed lines are the mean flows of each period.

The density plot of year 2008 flow data at Folsom 1 (Fig. 2) showed that the mode of runoff flow is lower before regulation compared to during regulation. The density plot of flow during regulation was more right skewed, showing the tendency of flows during regulation to be consistently lower values. The KS test of the flow distribution between before and during regulation of year 2008 at Folsom 1 resulted in a KS statistic = 0.34 (p < 0.001), which suggested that the runoff distributions were significantly different.

**Fig. 3:**
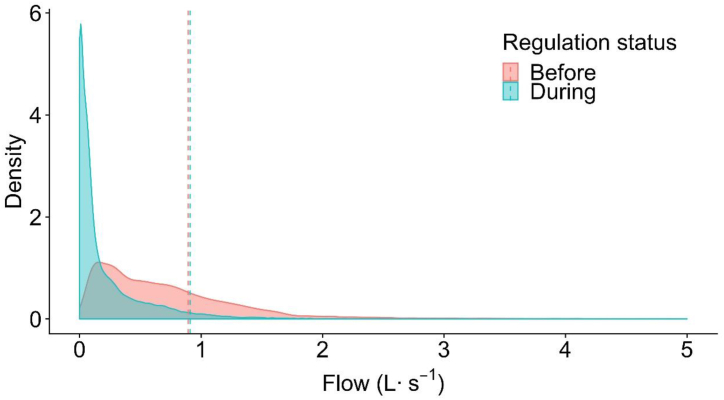
Density curve showing the relative frequency of runoff flow for 2008 at Folsom 2 before (June 1 – Aug. 27) and during (Aug. 28 – Oct. 28) landscape irrigation regulations. Density plot annual runoff for 2008 at Folsom 2. Vertical dashed lines are the mean flow of each period. In this case, the two dashed lines overlap each other.

**Fig. 4:**
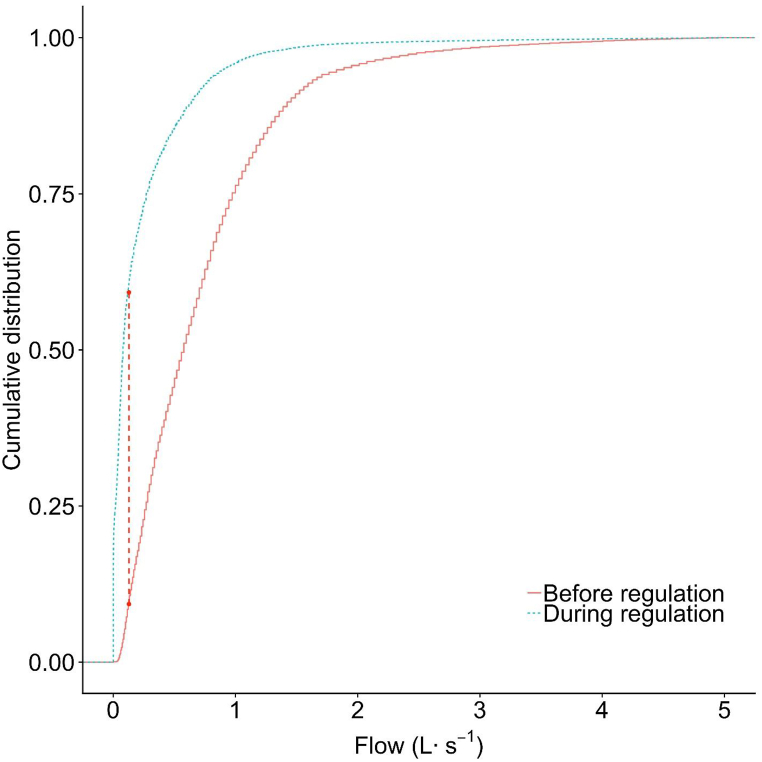
Illustration of the two-sample Kolmogorov–Smirnov (KS) statistic of drought 2008 at Folsom 2. Curves correspond to an empirical distribution function of the flows before (red solid) and during (blue dotted) the implementation of landscape irrigation regulation and the red dashed line is the two-sample KS statistic.

The Folsom 2 flow distribution during 2008 is shown as the density plot (Fig. 3). Although the mean flows overlapped each other in the density plot (Fig. 3), the previous *t*-test had shown that the transformed mean flows before and during regulation in 2008 were significantly different (p < 0.001) (Table 2). The flow during regulation was skewed more to the right and the mode of flow was smaller before regulation compared to during regulation, suggesting that the distribution of runoff between the two periods may be different. The initial slope of the Cumulative Distribution Function during regulation was much larger than before regulation (Fig. 4) and the frequency of lower flows was greater during the regulation. The KS test statistic was 0.5 (p < 0.001), suggesting that the runoff distributions were significantly different, as roughly 60 % of the runoff flows during regulation were less than 0.15 L s^−1^ compared to less than 10 % of runoff were below 0.15 L s^−1^ before regulation (Fig. 4).

**Fig. 5:**
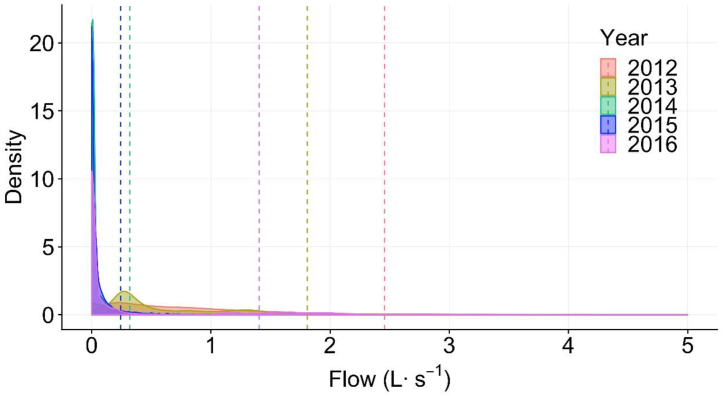
Density curve showing the relative frequency of runoff flow during 2012–2016 at Folsom 2. Years 2012 and 2013 were before implementation of landscape irrigation regulations and years 2014 and 2015 were during regulations. Year 2016 was after landscape irrigation regulations was lifted. Vertical dash lines are the mean flow of each year.

**Fig. 6:**
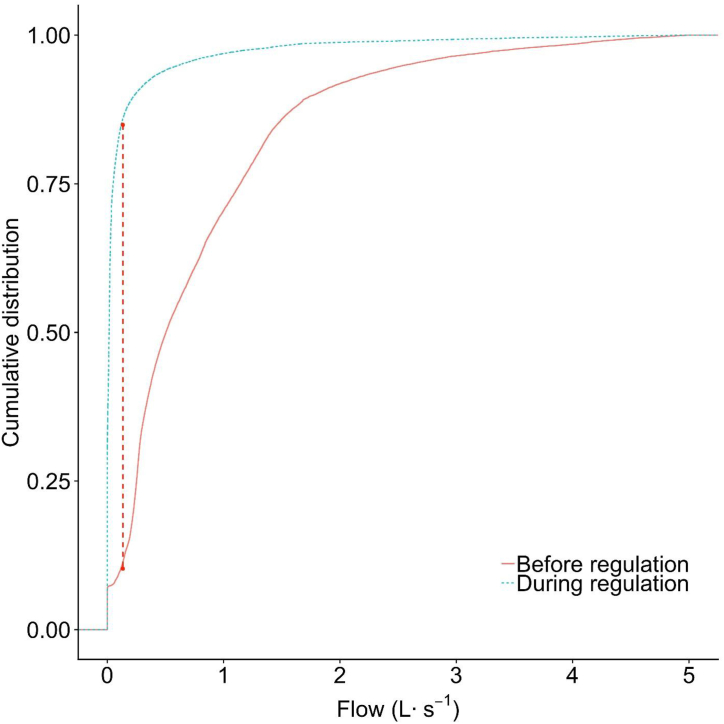
Illustration of the two-sample Kolmogorov–Smirnov (KS) statistic of the 2012–2016 drought in Folsom 2. Curved lines correspond to an empirical distribution function of the flow for before (red solid line) and during (blue dotted line) the implementation of landscape irrigation regulation and the red dashed line is the two-sample KS statistic Fig. 5c. Before regulations Fig. 5d. During regulations

The pattern of runoff changed during drought of 2012–2016 at Folsom 2 (Fig. 5). The mode of runoff flow was lower at the end of the drought (2016) compared to the earlier two years (2014–2015). The median annual flow continued to decrease during the sample period, from 2012 to 2015 (Table 4). Comparing flow distribution before and during regulation resulted in KS statistic = 0.738 (p < 0.001), which suggested that the runoff pattern changed significantly during the drought The Cumulative Distribution Function of these two periods of runoff data (Fig. 6) show more than 80 % of runoff data during regulation was smaller than 0.15 L s^−1^, compared to only 10 % of runoff data before regulation smaller than 0.15 L s^−1^.

The Statistic D, the absolute max distance between the Cumulative Distribution Function of two samples was determined for 2012–2015 at Folsom 2 (Table 5). The flow data distribution of each year is significantly different from that of the previous year as indicated by the KS test (p < 0.001) for each comparison. The closer statistic D is to 0, the more likely it is that the two samples were drawn from the same distribution. Statistic D increased as the duration of the drought increased, with the change in the flow distribution near the end of drought greater than the change in the distribution of the flow at the beginning of the drought.

**Table 5:** Two sample Kolmogorov-Smirnov test results of 2012–2015 runoff flows from Folsom 2. The null hypothesis of the Kolmogorov-Smirnov test was that samples from different years were drawn from the same distribution.

Year	Regulation status	Statistic D^a^	p-value^b^
**2012–2013**	Before - Before	0.118	***
**2013–2014**	Before - During	0.211	***
**2014–2015**	During - During	0.570	***

a. Statistic D = absolute max distance (supremum) between the Cumulative Distribution Functions of the two samples.

b. (***) Significant at p < 0.001.

### 3.3 Occurrence of runoff flows

**Fig. 7:**
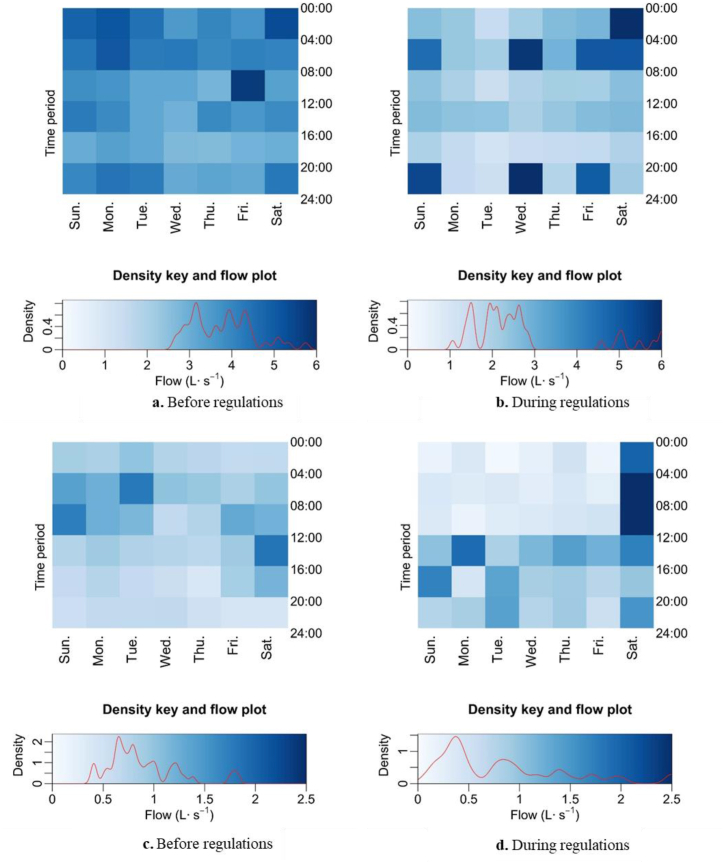
Heatmaps of mean flow for 4-h period during 2008 drought at Folsom 1 (7a and 7 b) and 2 (7c and 7 d) before (7a and 7c) and during (7 b and 7 d) implementation of landscape irrigation regulations. Fig. 7d does not include data from a storm on October 4, 2008. Magnitude of value to color is identified in the density key and flow plot. The red curve within the density key and flow plot represent the relative frequency of flows.

**Fig. 8:**
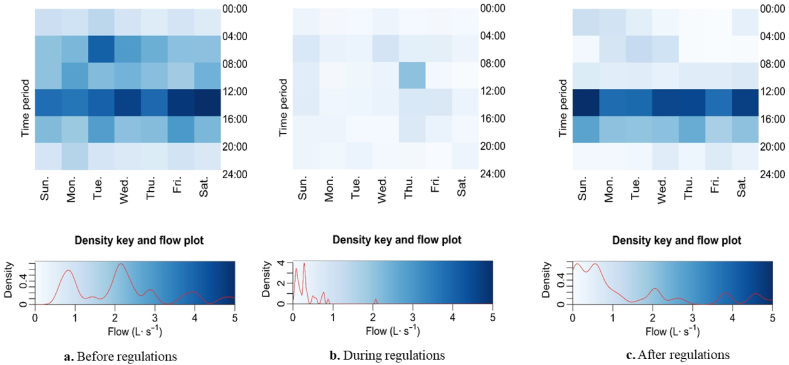
Heatmaps of mean flow for 4-h period during 2012–2016 drought at Folsom 2 before (8a), during (8 b), and after implementation of landscape irrigation regulations. Magnitude of value to color is identified in the density key and flow plot. The red curve within the density key and flow plot represent the relative frequency of flows.

Each square of the heatmap represents the mean flow within the associated 4-hr time period during that day of week (Fig. 7, Fig. 8). Darker blue color indicates higher mean flows while lighter color indicates lower flows. The range of flows were consistent within the same drought period and the key to the corresponding densities and flow plots are exhibited below the heatmap.

Based on the heatmaps of flows during 2008 at Folsom 1, flows between 2.5 and 4.5 L s^−1^ occurred before regulation (Fig. 7a). The majority of flows decreased to between 1 and 3 L s^−1^ during the watering regulation (Fig. 7b). A similar pattern was observed at the Folsom 2 (Fig. 7c and d) with a lower mean flow during the regulation. Runoff flows at both sites before regulation (Fig. 7a and c) were consistent throughout the day. Lower mean flows were observed during regulation (Fig. 7b and d) compared to before regulation were enacted. Folsom 2, both before and during regulation, had specific time periods associated with peak runoff. The cause of peak flow during regulation at Folsom 2, which occurred between 0:00–08:00 Saturday (data not shown), was attributed to an unusual storm event on October 4, 2008. After removing this event from the Folsom 2 data, the peak flow during regulation occurred between 12:00 and 16:00 on Monday at Folsom 2 (Figs. 7d) and 0:00 to 08:00 of Saturday at Folsom 1 (Fig. 7b).

Flows during the drought of 2012–2016 before regulation at Folsom 2 (Fig. 8a) suggested that the greatest runoff flows occurred between 12:00 and 16:00 and the distribution of flows during the day was distributed within the range of 0–3 L s^−1^. Flows during regulation (Fig. 8b) shows that, overall, flows were less than those prior to the drought and mainly fall within 0–1 L s^−1^. A slightly higher flow pattern can still be observed between 04:00 to 08:00 most days of the week. After watering regulation was lifted and the drought ended (Fig. 8c), the flow pattern resembled the pattern before regulation, as the flow between 0 and 2 L s^−1^ was nearly evenly distributed across the days of the week and the peak water runoff rates between 12:00 to 16:00 were more distinct than before. Additionally, flows greater than 3 L s^−1^ were significantly more frequent after the drought.

## 4 Discussion

As the majority of dry-season runoff is derived from landscape irrigation [[10], [15]], it is reasonable to assume that changes in mean runoff flows represent similar changes in landscape irrigation water use. In 2008, significant reductions of runoff flows were observed at both sites after watering regulation was implemented, suggesting that the regulations reduced dry season runoff. Flow distribution was largely influenced by the watering regulations, as runoff flows continually decreased after regulation was implemented. As the difference in mean runoff flows between 2013 and 2014 at Folsom 2 was 1.489 L s^−1^ when stage 3 watering regulation was implemented, a significantly larger difference (p < 0.001) than between other years (Fig. 1, Table 4), it can be concluded that long-term landscape watering regulation during drought could reduce urban runoff during dry periods, possibly by reducing the landscape water use that generates it.

Reductions in urban water runoff flows during the drought periods were accompanied by changes in daily flow patterns. Changes in flow patterns reflect a change in human management of landscape irrigation. A period of peak runoff flows occurred almost every day in a week before regulation was implemented (Fig. 7a, b, 7a). During the regulation, the median peak runoff flows occurred on less days of the week for both sites in 2008 (Fig. 7, Fig. 8b), suggesting that the change in flow pattern was in response to the Stage 2 irrigation regulation However, there is no peak shown in the heatmap of runoff during 2013–2014 when regulation was imposed (Fig. 8b). The overall mean runoff flow in 2013–2014 after Stage 3 regulation (Fig. 8b) was implemented was lower than the period before Stage 3 regulation was implemented (Fig. 8a) and was even lower than the flows when Stage 2 regulation was implemented in 2008 (Fig. 7b and d). Hayden et al. [19]saw no change in mean runoff volume from 2007 to 2008 at the same monitoring sites. However, that study did not separate 2008 into different periods based on drought regulations and the data analysis performed was “somewhat crude” and only intended to understand the effect of providing irrigation best management practice outreach to residences within the drainage [19].

Policy response to the drought may include investing in new water management technology and increasing enforcement against water waste to reduce municipal and agricultural water use. State policy aimed at reducing landscape water use only included a rebate program for replacing turf and a water-efficient landscape ordinance [20] that may not have been effective without imposing water regulation. When Governor Brown imposed the statewide 25 % reduction in potable urban water usage from April 2015 (State of California Executive Department, 2015), 44% of Californians said it would be difficult for them personally to make more of a sacrifice [21]. However, decrease in mean flows and the distribution of flows before and after regulation indicate that significant irrigation management changes occurred at the residential community level, driven by individual homeowners. Savings and efficiencies from individual implementation of household-scale water conservation efforts can accumulate into significant reduction in water use.

The drought in 2008 was a relatively localized event barely mentioned publicly or in the news. However, during the second longer and more extensive drought event, numerous news articles described dry conditions, water conservation measures, and severe drought consequences. This increase in media coverage resulted in drought awareness that permeated the public consciousness and influenced personal habits [22]. Of the two sites analyzed, Folsom 1 saw a significant decline in runoff volume while Folsom 2 saw an increase in runoff during 2008. However, during 2012–2015 there was significant reduction in runoff over multiple years at Folsom 2 and before watering regulation was imposed (Fig. 1). This supports previous research that households with higher level of drought awareness and concern have significantly lower level of irrigation water consumption [22,23].

The drought occurring in 2012–2015 was the single most arid period in California during the last 1200 years [24] and the mean runoff flow at Folsom 2 decreased significantly during that time (Fig. 1). Before the 2015 dry season, Governor Brown announced Executive Order B-29-15, a mandatory 25 % reduction in statewide water use [20]. Yet the lowest reduction in dry season urban runoff at Folsom 2 was between year 2014 and 2015, as it had the greatest statistic D (Fig. 1, Fig. 5, Table 5). The relatively small reduction in mean flow seen between 2014 and 2015 may be due to a greater amount of drought coverage by the media prior to Governor Brown's order which influenced residents to start applying water savings measures before implementation of the statewide regulation.

The heatmaps of the 2012–2015 (Fig. 8) period demonstrate that dry season runoff flow patterns, driven by residential irrigation management, changed during the drought. Consistent runoff reductions during formerly high water-demand periods is indicative of changes in residential irrigation water use. Management decisions that may have affected irrigation water use include reducing run time or even eliminating irrigation, incorporating low water-use plant material, reducing irrigation inefficiency, or car wash frequency. As Low et al. [25], found in Melbourne, Australia, many of these behaviors may persist after drought ends. However, increases in runoff were observed in 2016 at Folsom 2 after regulation was downgraded to Stage 1, indicating water-conserving behavior may have changed as soon as regulation was eased. Long-term regulation enacted outside of drought periods could assist in sustaining water conservation behaviors adopted during drought and enhance water-use efficiency.

Delaying the implementation of landscape water-use regulations until after a drought is fully underway might not be timely enough for future droughts. Other measures to consider before or at the onset of drought include stormwater runoff reuse and regulations on household water use Grant et al. [26]. Timely news and publicity about drought and water-use efficiency is needed to maintain focus on water conservation at the household level. Increased and long-term policy changes in water use and conservation practices are needed to affect landscape irrigation practices and reduce dry season urban runoff.

## 5 Conclusion

This study found that mean residential runoff flows decreased significantly during a long-term drought after landscape irrigation regulations were implemented. As runoff from residential areas is an indicator of irrigation water use, reductions in runoff signify similar reductions in irrigation water applied. This indicates that local landscape irrigation regulations could effectively reduce dry season urban runoff, even though it later rebounded after the 2012–2016 drought ended and regulation was eased. If landscape irrigation regulations implemented during drought periods remain in place after a drought ends, irrigation water use could remain low and decrease water waste. Although landscape irrigation regulations decrease runoff from residential landscapes, public awareness of the effects of severe drought may result in significant reductions in water use. Therefore, implementation of landscape irrigation regulations at onset of drought or continuously may be more effective than waiting until a drought occurs.

## Funding

This work was supported by the 10.13039/100004814California State Water Resources Control Board, 10.13039/100009503Sacramento, CA (Agreement No. 04-231-550-4) under a Proposition 50 CALFED Drinking Water Grant. The contents of this document do not necessarily reflect the views and policies of the State Water Board or CALFED, nor does mention of tradenames or commercial products constitute endorsement or recommendations for use.

## Data availability statement

Data has not been shared in a publicly available repository and will be made available upon request.

## CRediT authorship contribution statement

**Zhou Yang:** Writing – original draft, Visualization, Methodology, Formal analysis, Data curation. **Lorence R. Oki:** Writing – review & editing, Supervision, Resources, Project administration, Investigation, Funding acquisition, Conceptualization. **Jared A. Sisneroz:** Writing – review & editing, Investigation, Data curation, Conceptualization. **Darren L. Haver:** Writing – review & editing, Project administration, Funding acquisition. **Bruno J.L. Pitton:** Writing – review & editing, Writing – original draft, Supervision, Methodology, Investigation, Data curation, Conceptualization.

## Declaration of competing interest

The authors declare the following financial interests/personal relationships which may be considered as potential competing interests.
